# Placental Pathophysiology in Maternal Psychoactive Substance Use: Biological, Clinical, and Forensic Perspectives

**DOI:** 10.3390/cells15121128

**Published:** 2026-06-22

**Authors:** Oscar Fraile-Martinez, Natalia Rubio-Bedoya, Cielo García-Montero, Diego Liviu Boaru, Patricia de Castro-Martinez, Julia Bujan, Laura López-González, Raul Díaz-Pedrero, Natalio García-Honduvilla, Melchor Álvarez-Mon, Miguel A. Saez, Juan A. De León-Luis, Coral Bravo, Miguel A. Ortega

**Affiliations:** 1 Department of Medicine and Medical Specialities, (CIBEREHD), Faculty of Medicine and Health Sciences, University of Alcalá, 28801 Alcala de Henares, Spain; natalia.rubiob@edu.uah.es (N.R.-B.); cielo.garcia@uah.es (C.G.-M.); diego.boaru@edu.uah.es (D.L.B.); patriciadecastro1999@gmail.com (P.d.C.-M.); mjulia.bujan@uah.es (J.B.); natalio.garcia@uah.es (N.G.-H.); melchor.alvarezdemon@uah.es (M.Á.-M.); msaega1@oc.mde.es (M.A.S.); 2 Ramón y Cajal Institute of Sanitary Research (IRYCIS), 28034 Madrid, Spain; laura.lgonzalez@uah.es (L.L.-G.); raul.diazp@uah.es (R.D.-P.); 3 Department of Surgery, Medical and Social Sciences, Faculty of Medicine and Health Sciences, University of Alcalá, 28801 Alcala de Henares, Spain; 4 Immune System Diseases-Rheumatology and Internal Medicine Service, University Hospital Prince of Asturias, Networking Research Center on for Liver and Digestive Diseases (CIBEREHD), 28806 Alcala de Henares, Spain; 5 Pathological Anatomy Service, University Hospital Gómez-Ulla, 28806 Alcala de Henares, Spain; 6 Department of Public and Maternal and Child Health, School of Medicine, Complutense University of Madrid, 28040 Madrid, Spaincbravoarribas@gmail.com (C.B.); 7 Department of Obstetrics and Gynecology, University Hospital Gregorio Marañón, 28009 Madrid, Spain; 8 Health Research Institute Gregorio Marañón, 28009 Madrid, Spain

**Keywords:** placenta, psychoactive substances, pregnancy, placental toxicity, maternal-fetal well-being, forensic analysis, prevention, fetal development, prenatal diagnosis

## Abstract

Maternal psychoactive substance use during pregnancy represents a major threat to placental integrity and fetal development. As the central interface for maternal–fetal exchange, the placenta is highly susceptible to psychoactive substances, including alcohol, tobacco, cannabis, cocaine, opioids, and synthetic drugs, which can cross the placental barrier and induce structural and functional alterations. This review synthesizes current evidence on the biological mechanisms, diagnostic approaches, and forensic relevance of psychoactive substances-induced placental pathology. We summarize how different substances disrupt placental vascularization, oxidative balance, epigenetic regulation, and cellular viability, leading to impaired nutrient and oxygen transfer and increasing the risk of adverse outcomes such as intrauterine growth restriction, preterm birth, congenital anomalies, and long-term neurodevelopmental impairment. We further discuss the role of placental tissue in identifying prenatal drug exposure and reconstructing exposure timelines. Beyond its clinical relevance, placental examination provides objective evidence with potential forensic value in cases of suspected maternal substance use, while also informing non-punitive, evidence-based interventions. Overall, integrating placental pathology into reproductive health research and prenatal care offers a multidisciplinary framework to improve maternal–fetal outcomes and guide public health strategies addressing substance use during pregnancy.

## 1. Introduction

Drug use during pregnancy represents a growing public-health concern because of its adverse effects on fetal development and maternal health. Substance use during gestation may occur in women with a pre-existing substance use disorder as well as in those without prior history, reflecting a heterogeneous clinical spectrum that includes continued use, relapse, or *de novo* initiation during pregnancy [1,2]. During gestation, women undergo physical and psychological changes that may give rise to symptoms such as anxiety, irritability, or depression, which, together with other vulnerability factors, increase the likelihood of substance use [3]. However, these factors should be considered within a broader biopsychosocial framework rather than as primary determinants of substance use [4,5]. Substance use is one of the main preventable causes of disturbances in fetal development, and its detection in obstetric practice remains challenging due to time limitations, available tools, and specialized training [6,7].

Furthermore, the true prevalence of psychoactive substance use during pregnancy is likely underestimated when epidemiological studies rely exclusively on maternal self-report, questionnaires, or clinical interviews [8,9]. Disclosure may be limited by stigma, fear of social judgment, potential legal consequences, recall bias, and concerns about child-protection involvement [10]. Accordingly, several studies have shown discrepancies between self-reported prenatal exposure and objective detection through biological matrices, including urine, hair, meconium, umbilical cord tissue, and placental tissue [8,9,11,12]. This discrepancy is particularly relevant for alcohol, cannabis, tobacco/nicotine, and illicit substances, for which biomarker-based approaches may identify exposures that are not captured by conventional interviews [9,10,11,12,13,14]. Therefore, self-report and toxicological testing should be interpreted as complementary rather than mutually exclusive tools, especially when estimating prevalence, reconstructing exposure windows, or evaluating placental and neonatal consequences [15].

In addition, prenatal exposure to psychoactive substances frequently occurs in a polysubstance context, with overlapping use of alcohol, tobacco/nicotine, cannabis, opioids, cocaine, or synthetic drugs. This pattern is often underestimated in clinical and epidemiological studies and complicates both mechanistic attribution and clinical interpretation, since placental injury may reflect additive, synergistic, or mixture-specific effects rather than the action of a single compound [10,16,17,18,19].

The placenta is an organ formed during pregnancy that performs multiple functions throughout this period and constitutes a key structure for maternal–fetal well-being [20]. According to comprehensive global reviews, the prevalence of illicit drug use during pregnancy ranges dramatically from 1.65% when assessed through self-reported questionnaires to 12.28% when detected through toxicological analysis, indicating substantial underreporting in traditional surveillance methods [21]. Other epidemiological studies report that the data are even more marked in specific regions like Ethiopia or Brazil, where 22.0% and 18.28% of substance abuse during pregnancy has been defined in specific cohorts [22,23]. Tobacco, alcohol, and cannabis are the most abused substances during pregnancy, followed by cocaine and opioids [2]. Many of these agents can cross the placental barrier, exposing the fetus to concentrations similar to, or even higher than, those present in the mother [24]. This exposure has been associated with a variety of complications, including low birth weight, preterm birth, congenital malformations, and neurodevelopmental disorders, among other consequences [25].

Given the central role of the placenta in maintaining pregnancy and mediating maternal–fetal exchange, understanding how toxic substances can alter its structure and function is crucial from both medical and forensic perspectives. This paper focuses on the toxic effects of the main psychoactive substances of abuse on placental tissue, analyzing their mechanisms of action and the potential consequences for maternal–fetal health. In addition, special emphasis is placed on the forensic analysis of the placenta as a tool for detecting prenatal exposures, identifying specific patterns of damage, and its usefulness in judicial contexts related to negligence, abuse, or perinatal death.

## 2. Methodology

This work is based on a narrative review of scientific literature. The search was conducted in the databases PubMed, Scopus, and Web of Science (WOS), combining descriptors such as *placenta*, *pregnancy*, *psychoactive substance use*/*abuse*, *forensic analysis*, and *perinatal outcomes*. Boolean operators were used to refine the search and identify publications relevant to the objectives of this study.

We included articles published in English and Spanish, prioritizing original research papers, review articles, and clinical or forensic reports from peer-reviewed journals. While the primary focus was on literature published in the last 15 years, earlier studies were also considered when they provided fundamental mechanistic insights or addressed gaps not covered by more recent work.

The final selection of references was based on their relevance, methodological quality, and contribution to four key areas: (1) the biological role of the placenta and its vulnerability to toxic substances; (2) the effects of alcohol, tobacco, cannabis, cocaine, opioids, and additional classes of substances, including amphetamine-type stimulants (e.g., MDMA and methamphetamine), new psychoactive substances (e.g., synthetic cannabinoids and synthetic cathinones), and polysubstance use on placental tissue; (3) diagnostic and forensic applications of placental analysis; and (4) clinical, social, and legal implications.

This approach does not seek to systematically synthesize all available evidence, but rather to integrate and critically discuss the most significant findings that contribute to understanding the impact of maternal psychoactive substance use on the placenta and foetal health.

## 3. General Description of the Placenta and Its Importance in Pregnancy

The placenta is an organ formed from the embryo after the implantation process. Its development, known as placentogenesis, occurs in well-defined stages. Initially, a critical period called decidualization takes place, involving extensive morphogenetic, biochemical, and vascular remodeling of the maternal endometrium to facilitate implantation [20]. Following multiple cell divisions, the zygote develops into a blastocyst, which after implantation differentiates into two key structures: the inner cell mass, which will give rise to the fetus, and the trophectoderm, responsible for placental formation [26].

Within the trophectoderm, specialized cells known as trophoblasts progressively differentiate into distinct subtypes to perform essential placental functions [27]. After implantation, the syncytial fusion of mononucleated cells generates multinucleated syncytiotrophoblasts (STBs). These cells constitute the primary placental interface, separating maternal blood from fetal tissue, facilitating nutrient and gas exchange, and preventing the entry of harmful substances or immune cells [28]. Adjacent to the STBs but separated from maternal blood are the cytotrophoblasts (CTBs), regarded as progenitor cells of STBs, which play a fundamental role in the development of villous structures [29]. Together, STBs and CTBs constitute the fetal portion of the placenta, forming the chorionic villi. In addition, CTBs invade the maternal portion of the placenta (known as the decidua) and differentiate into extravillous trophoblasts (EVTs), which can be classified as: (1) Endovascular trophoblasts (eEVTs), which remodel the uterine spiral arteries, and (2) Interstitial trophoblasts (iEVTs), which remain in the decidua and perform essential functions [30].

During the first trimester, the placental structure is established as chorionic villi proliferate and branch. Primary villi, initially composed of STBs and CTBs, are invaded by extraembryonic mesodermal cells, forming a mesenchymal core and evolving into secondary villi [31]. By days 18–20, fetal capillaries appear within secondary villi, leading to the development of tertiary villi. These villi evolve into five distinct villous tree types, differing in developmental stage, structure, cellular components, vascular branching, and histological features [32,33], namely: (1) Mesenchymal villi: the initial form of all tertiary villi. They support villous proliferation and most placental endocrine activity. By term, they represent less than 1% of total villous volume, as they progressively differentiate into immature intermediate villi during the first and second trimesters and into stem villi in the third trimester. (2) Immature intermediate villi: an advanced stage of mesenchymal villi that serve as the main growth centers of villous trees. They dominate maternal–fetal exchange during the first and second trimesters, as terminal villi are not yet fully developed. (3) Stem villi: characterized by condensed fibrous stroma, large vessels, and microvessels. They act as the structural backbone of villous trees but do not participate in endocrine activity or direct maternal–fetal exchange. (4) Mature intermediate villi: show increased fetal vascularization, enabling efficient maternal–fetal exchange and leading to the formation of terminal villi. (5) Terminal villi: highly capillarized structures connected to stem villi through intermediate forms. They feature dilated sinusoids optimized for diffusive exchange and are fundamental for the transfer of oxygen, carbon dioxide, electrolytes, and nutrients between mother and fetus.

At birth, the mature placenta is composed of 15–28 subunits known as cotyledons, each forming a perfusion chamber supplied by one or more maternal spiral arteries and separated partially or completely by connective tissue [32]. Each cotyledon contains one or more fetal villous trees, including a fetal artery and vein. The placenta is bounded by the chorionic plate, a structure covered by the amnion that serves as the anchoring site for the umbilical cord, and by the basal decidual plate in contact with the endometrium.

The cytoarchitecture of the placenta comprises the following components:

- Fetal portion of the placenta (Chorion frondosum): composed of STBs, primarily responsible for maternal–fetal exchange, and CTBs, which regenerate STBs and EVTs. Additional cells derived from mesenchymal differentiation can also be identified, such as endothelial cells, fibroblasts, myofibroblasts, smooth muscle cells, and macrophages (Hofbauer cells). Fetal vessels within villous trees include capillaries and sinusoids in terminal villi, as well as arteries, arterioles, veins, and venules in stem and intermediate villi, whose regulation is mediated by autocrine and paracrine mechanisms [34].
- Maternal portion of the placenta (Basal decidua): Composed of trophoblastic cells, including iEVTs and eEVTs, essential for placentation and immune modulation, influencing decidual natural killer cells (dNKs), macrophages, and T cells [35].
- Fibrinoids: extracellular deposits within placental tissue, including [36].
  - Fibrin-type fibrinoids: composed of fibrin and products of maternal blood coagulation that support villous tree growth and blood flow.
  - Matrix-type fibrinoids: secreted by EVTs, facilitating trophoblastic invasion through interactions with cell-surface integrins.

Thus, the placenta is a fundamental organ that establishes the connection between mother and fetus during gestation, allowing the exchange of nutrients, oxygen, and waste products. It also plays an essential role in fetal metabolism, using oxygen and glucose for energy production and processing nutrients required for development [37]. In addition, it functions as an endocrine center, synthesizing key hormones necessary for pregnancy maintenance, such as human chorionic gonadotropin (hCG), estrogens, and progesterone, which support both fetal development and maternal adaptation to gestation [20]. The placenta also secretes leptin and adiponectin, which regulate maternal–fetal metabolism, as well as PP13 protein, a determinant in the regulation of placental blood flow. Furthermore, the placenta acts as a protective filter, preventing the transmission of infections and neutralizing potentially toxic substances through detoxifying enzymes. For example, under maternal stress or exposure to harmful substances such as tobacco, the enzyme 11β-HSD2 is activated, converting cortisol into cortisone and thereby reducing its negative impact on fetal development [38]. Finally, the placenta plays a crucial role in fetal programming, a process by which intrauterine conditions can influence long-term health. Maternal nutrition during gestation may condition the newborn’s predisposition to various pathologies and physiological traits [20]. Moreover, inadequate blood flow between the uterus and placenta is associated with an increased risk of cardiovascular diseases later in life [39].

**Figure 1:**
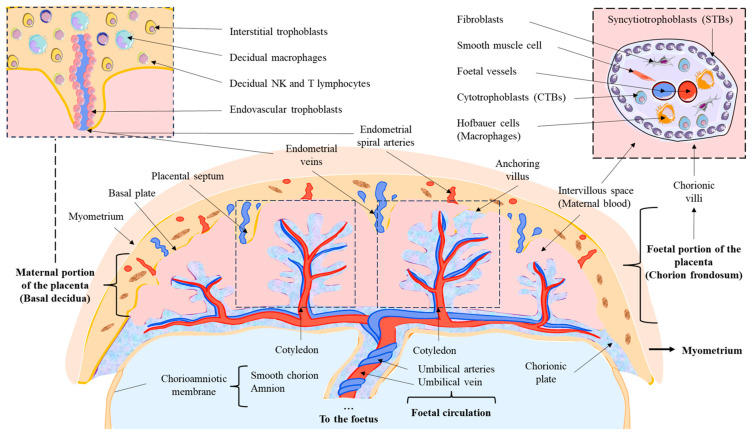
Placental structure with their main components.

In Figure 1, the structure and associated cells from the placenta are summarized.

## 4. Placental Examination in Maternal Psychoactive Substance Use

### 4.1. Clinical and Pathophysiological Relevance of Placental Examination

Maternal psychoactive substance use exerts significant effects on fetal development, many of which can be identified through placental examination. As a highly specialized and dynamic interface between mother and fetus, the placenta not only reflects systemic maternal exposures but also constitutes a direct biological target of xenobiotic-induced injury. Consequently, placental analysis provides a unique opportunity to establish mechanistic links between maternal psychoactive substance use and adverse fetal outcomes, integrating clinical, histopathological, and toxicological evidence [40]. This dual role—both as a biomarker of exposure and as a mediator of pathological processes—positions the placenta as a central element in the evaluation of substance use during pregnancy.

At the tissue level, maternal substance use is associated with distinct patterns of placental injury that compromise fetal development. Substances such as cocaine, cannabis, amphetamines, and opioids have been shown to induce vascular, inflammatory, and structural alterations that impair the placenta’s capacity to ensure adequate oxygen and nutrient exchange [41]. Among the most relevant histopathological findings are maternal and fetal vascular malperfusion, placental infarctions, intervillous thrombi, and abnormalities in villous maturation, all of which reflect underlying placental insufficiency and are strongly associated with intrauterine growth restriction, low birth weight, and preterm birth [42,43]. Structural alterations such as reduced placental size, thrombotic lesions, and signs of hypoperfusion further reinforce the concept of the placenta as a primary target of psychoactive substance-induced toxicity [44,45].

In addition to histopathological evidence, extensive literature supports the association between prenatal exposure to substances and both placental dysfunction and fetal pathology. Alcohol consumption during pregnancy is strongly linked to fetal alcohol spectrum disorders, characterized by growth restriction, neurodevelopmental impairment, and structural abnormalities [46]. Tobacco use has been consistently associated with placental insufficiency, low birth weight, and increased perinatal morbidity, likely mediated through vascular and hypoxic mechanisms [47]. Illicit drugs such as cocaine and cannabis have been associated with vascular disruption, congenital malformations, and long-term neurobehavioral alterations [48,49]. Similarly, the use of medications with known teratogenic potential—particularly in the absence of medical supervision—can result in severe developmental defects, further highlighting the importance of monitoring drug exposure during pregnancy [50]. Notably, many of these effects are mediated, at least in part, through placental injury, reinforcing its central role in the pathogenesis of fetal damage.

Placental examination plays a critical role in elucidating the causes of stillbirth and neonatal death, particularly when maternal drug exposure is suspected. When combined with fetal autopsy and maternal clinical history, it allows identification of key pathological processes such as hypoxia, infection, and vascular compromise, which frequently underlie adverse perinatal outcomes [51,52]. Observational studies have consistently demonstrated that placental pathology contributes to a substantial proportion of fetal deaths, often associated with vascular dysfunction or inflammatory lesions [53]. These findings are especially relevant in the context of substance use, where placental dysfunction represents a major mechanistic pathway linking maternal exposure to fetal morbidity and mortality.

### 4.2. Forensic and Medico-Legal Relevance of Placental Examination

Maternal psychoactive substance use during pregnancy is also embedded within a complex social and legal context that directly influences clinical management, healthcare access, and the interpretation of biological evidence. Across jurisdictions, approaches range from non-coercive, treatment-oriented models to punitive frameworks that criminalize substance use during pregnancy. In most European countries, policies emphasize harm reduction and access to care, although notable exceptions exist, such as Norway, where legislation permits involuntary admission of pregnant women when substance use poses a significant risk to fetal health [54,55]. In contrast, countries such as Canada and New Zealand prioritize maternal autonomy and limit coercive interventions [56]. More restrictive approaches have been implemented in other regions, particularly in the United States, where prenatal psychoactive substance exposure may be legally classified as child abuse in several states [57]. Evidence suggests that such punitive policies may discourage women from seeking prenatal care, thereby increasing maternal and fetal risk [6,58,59], whereas integrated and non-punitive care models combining obstetric follow-up, addiction treatment, and psychosocial support have been associated with improved perinatal outcomes [60,61].

From a forensic perspective, placental examination can provide objective evidence in cases of suspected prenatal psychoactive substance exposure, stillbirth, neonatal death, neglect, or disputed perinatal causality. The placenta may serve both as a biological matrix for toxicological testing and as a tissue record of exposure-related injury, helping to integrate evidence of exposure with pathological mechanisms potentially contributing to fetal or neonatal harm. This is particularly relevant when maternal self-report is incomplete or unavailable, when exposure is denied, or when biological evidence is required to reconstruct the clinical and toxicological context of pregnancy.

A key advantage of placental analysis lies in its ability to support the assessment of the timing and extent of fetal exposure to drugs. Establishing the chronology of exposure is essential for determining causality, particularly in medico-legal contexts. Placental tissue, together with complementary biological matrices such as umbilical cord, meconium, or neonatal hair, provides objective evidence of in utero substance exposure, each with distinct detection windows and limitations [62]. While meconium and umbilical cord tissue are widely used due to their accessibility and analytical sensitivity, the placenta offers a valuable record of exposure, particularly during the second and third trimesters, and allows direct correlation between toxicological findings and histopathological alterations [62]. Importantly, the impact of substance exposure varies according to gestational stage, with early exposure more closely associated with teratogenic effects, and later exposure linked to growth restriction, preterm birth, and functional impairment [40].

However, the clinical and forensic use of such evidence requires careful interpretation, as its application in punitive legal frameworks may reinforce stigma, bias, and healthcare avoidance. Therefore, strict adherence to chain-of-custody standards and a non-punitive, health-centered approach are essential to ensure that placental findings support clinical decision-making rather than contribute to adverse social consequences [63]. Equally important, the use of such evidence should avoid reinforcing stigma, bias, or healthcare avoidance; therefore, a non-punitive, health-centered approach is essential to ensure that placental findings support clinical decision-making and maternal–fetal care rather than adverse social consequences.

**Figure 2:**
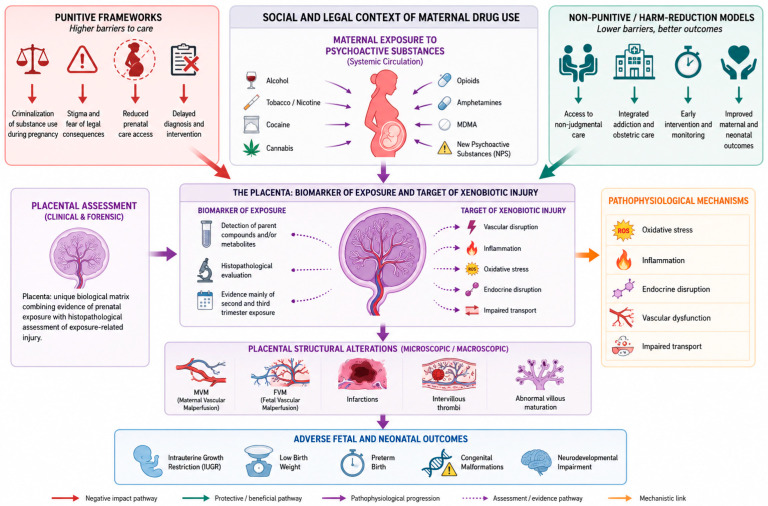
Placenta-centered framework linking maternal psychoactive substance use to adverse fetal and neonatal outcomes. The figure illustrates a multidimensional model in which maternal psychoactive substance use is shaped by the surrounding social and legal context, contrasting punitive approaches, which may reduce prenatal care access and delay diagnosis, with non-punitive and harm-reduction models, which favor early intervention and improved outcomes. At the center of the framework, maternal exposure to substances—including alcohol, tobacco, cocaine, cannabis, opioids, amphetamines, MDMA, and new psychoactive substances (NPS)—converges on the placenta, represented as both a biomarker of exposure and a target of xenobiotic injury. From a clinical and forensic perspective, placental tissue is highlighted alongside other biological matrices used to assess prenatal exposure, particularly because it can provide information on second- and third-trimester exposure while also enabling histopathological evaluation. The figure further summarizes key pathophysiological mechanisms triggered by psychoactive substance exposure, including oxidative stress, inflammation, endocrine disruption, vascular dysfunction, and impaired transport, which may lead to characteristic microscopic and macroscopic placental alterations such as maternal vascular malperfusion (MVM), fetal vascular malperfusion (FVM), infarctions, intervillous thrombi, and abnormal villous maturation. These placental abnormalities ultimately contribute to adverse fetal and neonatal outcomes, including intrauterine growth restriction (IUGR), low birth weight, preterm birth, congenital malformations, and neurodevelopmental impairment. Note: This figure was performed by providing specific prompts developed by the authors to the Platform FigureLabs (available online) and modified by using ChatGPT5 (GPT-5, OpenAI, San Francisco, CA, USA). The authors have reviewed and edited the output and take full responsibility for the content of this figure.

In Figure 2, we summarize a placenta-centered conceptual framework linking maternal psychoactive substance use to adverse fetal outcomes across multiple levels of analysis. The figure integrates the social and legal context of substance use during pregnancy with maternal exposure patterns, placental toxicological and histopathological assessment, underlying pathophysiological mechanisms, and the resulting structural and developmental consequences. Understanding this continuum—from social and legal determinants to biological mechanisms and structural placental alterations—provides a comprehensive framework for interpreting the impact of maternal psychoactive substance use on fetal development. This integrative perspective supports the analysis of the molecular and cellular mechanisms by which specific substances disrupt placental function, which are explored in detail in the following section.

## 5. Effect of Psychoactive Substances on Placental Tissue

### 5.1. Alcohol

Alcohol (ethanol) is, with tobacco, one of the most commonly consumed psychoactive substances during pregnancy [64]. Global epidemiological studies estimate that 10% of pregnant women consumed alcohol. In Europe, 16% of women resident in Europe report alcohol consumption during pregnancy, with the highest rates in UK (28.5%), Russia (26.5%) and Switzerland (20.9%) [65]. On the other hand, a downward trend in alcohol consumption among pregnant women has been observed in recent years [66,67]. Alcohol use tends to be higher in the first but is generally reduced in the second and third trimesters [68]. Howsoever, alcohol intake during pregnancy alters placental function through mechanisms that compromise fetal development. Ethanol readily crosses the placenta and accumulates in the fetus at concentrations similar to or higher than maternal levels, owing to the immaturity of fetal metabolic systems [69].

Alcohol exposure can induce microinfarctions in the placenta, reduce blood flow and oxygenation, and, although the placenta retains some capacity for adaptation during early stages, placental injury may persist and impair fetal growth [70,71]. The principal mechanism by which alcohol disrupts fetal growth is hypoxia, as it decreases oxygen availability, interferes with nutrient transport, and impairs placental protein synthesis [70,72]. In addition, alcohol exposure triggers massive apoptosis in trophoblastic cells through activation of calcium-dependent signaling cascades, requiring both intracellular calcium mobilization and extracellular calcium influx mediated by TRPC channels [73]. These changes disrupt placentation and directly contribute to IUGR, a hallmark of fetal alcohol spectrum disorders (FASD), whose manifestations vary depending on timing, dosage, and fetal sex [71,74].

The STB, responsible for maternal–fetal nutrient transfer, is particularly vulnerable to ethanol. Its membrane regulates transport and enzymatic activity; alterations in membrane fluidity can affect fetal glucose and sodium supply [72]. Alcohol specifically interferes with the active transport of essential amino acids across the placenta, significantly decreasing plasma levels of threonine, serine, glutamine, glycine, alanine, methionine, and proline in both maternal and fetal compartments. Notably, histidine transport is reduced by 41–51% in the fetus, impairing central nervous system development due to its essential role in neurogenesis [75].

Alcohol also alters the expression of genes and proteins related to placental growth and function; it downregulates vascular growth factors such as PIGF and VEGFR2, decreases protein synthesis and iodine content, and modifies the expression of alcohol-metabolizing enzymes [76,77]. Furthermore, transcriptomic studies have identified dysregulation of genes involved in erythropoiesis, angiogenesis, and extracellular matrix organization [3,78]. Alcohol exposure also triggers an inflammatory response characterized by elevated levels of proinflammatory cytokines such as IL-1β, TNF-α, IL-6, MCP-1, and fractalkine in maternal serum, amniotic fluid, and placental tissue—mediated by TLR4 activation, which perpetuates inflammation and compromises fetal neurodevelopment [79,80].

Following ethanol exposure, placental energy metabolism may shift from carbohydrate to lipid utilization, contributing to reduced fetal brain weight—one of the characteristic features of FASD [78,81]. In perfused human placental villi exposed to ethanol, significant increases in nitrotyrosine (a marker of oxidative stress) and 8-hydroxyguanosine (8-OHdG, a marker of oxidative DNA damage) have been observed within just two hours of exposure [82]. These rapid effects coincide with mitochondrial dysfunction, evidenced by reduced expression of cytochrome c oxidase and ATP synthase [83]. Alcohol induces severe mitochondrial impairment characterized by decreased expression of electron transport chain complexes I and II (the primary entry sites), with compensatory upregulation of complexes III, IV, and V to sustain ATP production. This mitochondrial alteration is accompanied by mitochondrial DNA damage, oxidative stress, and activation of p21-, Bax-, and Bak-dependent apoptotic pathways through p53-independent mechanisms [84,85].

Additionally, alcohol induces endoplasmic reticulum (ER) stress in placental tissue—albeit less pronounced than that caused by nicotine—activating markers such as GRP78/BiP and IRE1α, which may interfere with essential placental functions and compromise fetal growth and development [86]. ER stress contributes to the neurodegenerative component of FASD through accumulation of misfolded proteins and activation of programmed cell death pathways [87].

Importantly, alcohol affects the placenta in a sex-dependent manner. A human cohort study revealed that first-trimester alcohol exposure reduces global DNA methylation in male placentas, associated with decreased placental thickness and reduced levels of placental growth factor (PGF) [88]. In contrast, female placentas exhibit increased levels of S-adenosylmethionine (SAM), a methyl donor, and overexpression of TET3, an enzyme involved in active DNA demethylation. This divergence suggests sex-specific compensatory mechanisms to counteract alcohol-induced stress.

Collectively, these findings highlight the placenta as a dynamic sensor of prenatal alcohol exposure, integrating morphological, epigenetic, and functional responses that shape the risk of developing FASD.

### 5.2. Nicotine and Tobacco Components

Tobacco is the most widely consumed substance during pregnancy, affecting a large number of gestations due to its high prevalence. Most women who smoke begin before the age of 26, and a considerable proportion continue smoking beyond 20 weeks of gestation. Tobacco use is nearly universal among pregnant women with a history of substance use [89].

Prenatal tobacco exposure induces significant morphological changes in placental tissue, compromising both its function and fetal development [90]. Nicotine, the main addictive component of tobacco, crosses the placenta and accumulates in amniotic fluid as well as in fetal and placental tissues, reaching concentrations that can exceed maternal levels [91]. This exposure leads to chronic fetal hypoxia and uteroplacental vasoconstriction, predisposing to placental insufficiency and IUGR [92,93].

Histopathologically, nicotine induces structural alterations such as STB hyperplasia, excessive formation of syncytial knots, abnormal cytotrophoblast proliferation, necrotic areas in the syncytial layer, and irregular folding of the outer membrane—reflecting tissue injury and cellular stress [94,95]. Maternal tobacco exposure during the first trimester significantly increases the expression of pro-apoptotic genes (TP53, BAX) and the BAX/BCL2 ratio, indicating enhanced trophoblastic apoptotic potential that may compromise early placentation. This apoptotic process is accompanied by villous hypoxia that influences both angiogenesis and programmed cell death. Increased neutrophil infiltration has also been observed, marked by elevated neutrophil gelatinase–associated lipocalin (NGAL) and overexpression of inflammatory genes such as IL8, CXCL10, S100A8, and S100A9, which recruit immune cells and perpetuate placental inflammation [96].

However, nicotine-induced inflammation displays a paradoxical duality. On one hand, nicotine exerts anti-inflammatory effects via activation of the α7 nicotinic acetylcholine receptor (α7nAChR) expressed in placental cells, suppressing proinflammatory cytokines (TNF-α, IL-6, IL-1β, MCP-1) through inhibition of the NFκB pathway [97,98,99]. This mechanism has been associated with a lower incidence of preeclampsia among smokers [97]. On the other hand, maternal smoking significantly increases neonatal IL-8 levels, suggesting fetal immune programming toward a proinflammatory phenotype predisposing to respiratory and allergic disease [100]. Moreover, maternal smoking disrupts decidual macrophage polarization, shifting the balance toward a proinflammatory M1 phenotype at the expense of the anti-inflammatory, reparative M2 phenotype. This is due to reduced α7nAChR expression in decidual macrophages, leading to increased TNF-α, IL-1β, and iNOS production, and reduced IL-10 and arginase-1 synthesis [101]. Simultaneously, maternal smoking dampens Toll-like receptor (TLR)-mediated innate immune responses in neonates, decreasing cytokine production (TNF-α, IL-6, IL-10) in response to TLR-2, TLR-3, TLR-4, and TLR-9 ligands—potentially predisposing to infection and immune dysregulation [102]. This duality may stem from nicotine’s local anti-inflammatory actions in the placenta being outweighed by the systemic oxidative and inflammatory effects of tobacco smoke.

Nicotine directly impairs the STB’s capacity for maternal–fetal exchange and alters the expression of structural and transport proteins, including reduced expression of Synb and nucleoside transporters such as hCNT1 [95,103,104]. It interferes with the active transport of essential amino acids, suppressing amino acid uptake by placental villi and significantly lowering amino acid levels in both placental tissue and the umbilical vein [105,106]. Similar effects have been observed with nicotine-containing electronic cigarettes compared to nicotine-free ones, mainly due to reduced efficiency of placental transporters, thereby impairing fetal amino acid supply [107]. This interference occurs through blockade of cholinergic receptors that mediate acetylcholine-dependent amino acid transport, particularly affecting arginine and system A amino acids [108]. As a compensatory mechanism, new amino acid transport systems are upregulated—such as those for α-aminoisobutyric acid uptake—although this compensation is insufficient to prevent fetal nutrient deficiency [105].

The expression and localization of nicotinic acetylcholine receptors (nAChRs) in STB, cytotrophoblast (CTB), and fetal endothelium indicate their functional role in cholinergic signaling and placental transport—processes disrupted by nicotine exposure [93,103]. Nicotine-induced placental dysfunction may also involve epigenetic alterations such as DNA hypermethylation of key placental genes [93,96], and dysregulation of specific microRNAs, notably downregulation of miR-16, miR-21, and miR-146a in placental tissue from smoking mothers [109]. Reduced miR-146a expression is particularly relevant, as it increases TRAF6-mediated NFκB signaling, promoting abnormal cell survival and contributing to tobacco-related placental pathology [110].

Tobacco exposure elevates placental oxidative stress, evidenced by increased lipid peroxidation markers (4-hydroxynonenal) and DNA oxidation (8-hydroxy-2′-deoxyguanosine), as well as upregulation of the phase I enzyme CYP1A1 [109]. Cotinine, a nicotine metabolite, accumulates in placental tissue and serves as a reliable biomarker of fetal exposure, correlating with the induction of xenobiotic-metabolizing enzymes such as CYP1A1 and CYP1B1 in fetal tissues [111]. Maternal smoking also significantly reduces total placental antioxidant capacity, shifting the redox balance toward oxidative dominance [112]. This is associated with increased apoptosis, as evidenced by positive TUNEL staining in placentas of smoking mothers, and is reflected in amniotic fluid by lower antioxidant capacity and higher oxidative markers [109].

Nicotine also induces ER stress in placental tissue through activation of nicotinic acetylcholine receptors, triggering PERK and eIF2α phosphorylation, leading to global inhibition of protein translation and activation of the unfolded protein response [113,114]. This ER stress is linked to impaired disulfide bond formation via reduced expression of protein disulfide isomerase (PDI) and QSOX1, compromising proper protein folding and contributing to placental insufficiency [115]. Furthermore, nicotine induces autophagy in placental cells, a process that may disrupt placental homeostasis and contribute to pregnancy complications associated with maternal smoking [116].

In conclusion, tobacco use during pregnancy remains highly prevalent and exerts profound, multifactorial effects on the placenta, compromising its structure, function, and ability to sustain healthy fetal development.

### 5.3. Cannabis

Cannabis, or marijuana, is a plant whose dried parts—such as leaves, stems, flowers, and seeds—are used for various purposes. It contains more than 100 different compounds, with tetrahydrocannabinol (THC, used recreationally) and cannabidiol (CBD, used medically) being the most common [117,118]. It is estimated that between 3% and 16% of pregnant women worldwide use cannabis during pregnancy, although this percentage may be higher in some demographic subgroups [119].

This substance can alter placental tissue both histopathologically and structurally by reducing the placenta’s ability to transport blood and nutrients, thus mimicking the effects of placental insufficiency and inducing abnormalities in the expression of genes related to blood vessel development [120,121]. This alteration is partly due to the disruption of the endocannabinoid system (ECS), which plays a crucial role in implantation and placental development. The balance of this system becomes compromised with exogenous cannabinoid exposure, potentially affecting placental growth and function [120,122].

With regard to the STB, THC exerts a dual effect: at low doses, it can protect cells through its antioxidant action, but at high doses or with prolonged exposure, it can induce cellular apoptosis or decrease cell viability, as well as hinder trophoblast fusion and differentiation, thereby impairing hormone production such as human chorionic gonadotropin (hCG) and leptin [123,124]. At the molecular level, THC induces severe mitochondrial dysfunction characterized by reduced expression of mitochondrial biomarkers such as HSP60 and HSP70, increased markers of mitochondrial fission, and impaired cellular respiration, coinciding with reduced BeWo cell fusion and lower secretion of key fetal growth factors such as human placental lactogen (hPL) and IGF2 [125]. These effects are mediated in part via CB1 receptor activation in syncytialized BeWo cells. In parallel, THC directly induces ER stress through dose-dependent activation of CB1R/CB2R receptors, increasing all major ER stress markers including CHOP—effects that can be blocked by cannabinoid antagonists. This ER stress leads to aberrant placental gene expression and impaired mitochondrial function [126].

Similarly, cannabis consumption alters placental protein composition and molecular components, as both THC and CBD modulate the expression of key genes and proteins such as aromatase (CYP19), PP13, and breast cancer resistance protein (BCRP). These changes affect hormone production—including estradiol, β-hCG, and leptin—and increase placental barrier permeability, exposing the fetus to xenobiotics and pharmaceuticals [124,127]. CBD, in particular, significantly impairs angiogenesis and vascular formation, downregulating biological processes such as tube morphogenesis, blood vessel development, and vascular growth. Placentas exposed to CBD also show altered expression of glucose transporters (decreased GLUT1 and GR, increased GLUT3) and cellular distribution shifts, especially in syncytiotrophoblast layer II and endothelial cells [128].

Minor cannabinoids such as cannabidivarin (CBDV) and cannabigerol (CBG) also exert deleterious effects by inducing TRPV1-dependent ER stress in extravillous trophoblasts, differentially activating the unfolded protein response (UPR): both cannabinoids engage the IRE1 pathway, while only CBDV enhances downstream PERK effectors including p-eIF2α, ATF4, and CHOP [129]. These compounds also upregulate angiogenic factors such as VEGFA, PGF, and sFLT1, while disrupting endothelial-like behavior of HTR-8/SVneo cells and reducing tube formation.

Cannabis exposure during pregnancy also triggers a complex inflammatory response characterized by the suppression of immune gene networks in the placenta. Both THC and CBD upregulate four key inflammatory cytokines and chemokines (IL-1β, RANTES, TNFα, and IL-6) [130]. This inflammatory state correlates with the activation of natural killer (NK) cells and macrophages, where NK cell infiltration has been associated with fetal resorption due to their cytotoxic effects on trophoblast proliferation [131]. The suppression of immune-related genes in placentas from cannabis users may impair antiviral defense mechanisms and disrupt key processes of successful placentation, including trophoblast invasion and establishment of maternal-fetal blood flow [132,133].

At the epigenetic level, prenatal THC exposure is associated with significant alterations in DNA methylation in both placental and fetal tissues. In rhesus macaque placentas, 573 differentially methylated CpG sites were identified (335 hypermethylated, 238 hypomethylated), with the most significant site (cg23018092) located in the MEGF10 gene, showing hypermethylation following THC exposure [134]. In humans, prenatal cannabis exposure has been linked to genome-wide differences in DNA methylation detectable at ages 0, 7, 15–17, and 27 years, particularly in genes and pathways associated with neurodevelopment [135].

Overall, these findings indicate that placental function can be significantly altered by cannabis exposure, compromising maternal-fetal blood and nutrient transport, and affecting critical processes such as trophoblast formation and function. These disruptions may lead to severe fetal developmental consequences, particularly regarding neurodevelopment [120,123].

### 5.4. Cocaine

Cocaine is one of the most widely consumed stimulant drugs globally, available in several forms such as cocaine hydrochloride powder and crack cocaine. Its primary mechanism of action involves blocking dopamine (DA) reuptake, resulting in intense stimulation of the central nervous system (CNS) [136]. While national surveys in the U.S. have estimated the prevalence of cocaine use during pregnancy at approximately 1.1%, cohort studies employing biological analysis methods, such as meconium testing, have reported significantly higher rates, reaching 31%, whereas other surveys have estimated that 10% of pregnant women using illicit drugs during pregnancy consume cocaine [137].

In placental tissue, cocaine induces vasoconstriction by blocking presynaptic catecholamine receptors, thereby elevating circulating levels of adrenaline and noradrenaline. This triggers activation of the sympathetic nervous system (SNS), leading to marked placental vasospasm, hypertension, and increased uterine contractility, which collectively reduce placental blood flow [138,139]. This vasospasm also results from increased endothelin (a potent vasoconstrictor) and decreased nitric oxide (NO) (a vasodilator) production. These functional alterations are compounded by structural damage to the placental vasculature, amplifying their pathological significance [140].

Cocaine damages endothelial cells, increases vascular permeability to low-density lipoproteins (LDL), promotes leukocyte adhesion, and enhances platelet aggregation, all of which can contribute to adverse pregnancy outcomes [140]. Owing to its lipophilic nature and low molecular weight, cocaine rapidly crosses the placenta via simple diffusion, reaching maternal-placental equilibrium within minutes [141,142]. In fact, a systematic review found that cocaine and its metabolites, such as benzoylecgonine and norcocaine, are stored in the myometrium and placental membranes, maintaining continuous drug delivery to the amniotic fluid and exerting prolonged toxic effects on fetal development [143].

Neither cocaine nor its metabolites bind to plasma proteins, and although they have a relatively short plasma half-life, they can persist longer in placental tissue due to reduced plasma cholinesterase activity during pregnancy. This slows their degradation, enhances local toxicity, and allows the accumulation of both cocaine and its active metabolite norcocaine [142,144]. Furthermore, the cardiovascular adaptations of pregnancy, including increased blood volume and decreased vascular resistance, may potentiate cocaine’s vasoconstrictive effects. Notably, even umbilical arteries, despite lacking sympathetic innervation, have been shown to contract in response to cocaine exposure [142].

Because cocaine is eliminated more slowly from placental tissue, its local toxicity increases, reducing placental blood flow and oxygenation. The placenta therefore fails to act as an effective barrier to the transfer of cocaine and its metabolites [145]. Histopathological examinations of placentas exposed to cocaine reveal villous hemorrhage and edema of the chorionic villi, though fetal vasoconstriction is not always observed, suggesting that these effects are mediated primarily by maternal vascular mechanisms [146].

Cocaine exposure induces massive oxidative stress in placental tissue, as evidenced by significant alterations in the antioxidant enzymes catalase, superoxide dismutase, and glutathione peroxidase, leading to increased reactive oxygen species (ROS) and lipid peroxidation [147,148]. Although not directly studied in placental mitochondria, cocaine-induced oxidative stress is associated with mitochondrial dysfunction, including reduced activity of respiratory chain complexes, particularly Complex I (NADH dehydrogenase), which compromises ATP production and exacerbates tissue hypoxia [149]. Cocaine can also trigger cellular apoptosis by activating caspases (especially caspase-3) and disrupting the balance between pro-apoptotic (Bax) and anti-apoptotic (Bcl-2) proteins, a process that may persist even after exposure cessation [150].

At the molecular transport level, cocaine functions as a high-affinity competitive inhibitor of the placental serotonin transporter (Ki ≈ 0.09 μM) and also inhibits the placental norepinephrine transporter (Ki 1–10 μM), though it is not a transportable substrate [151]. Cocaine specifically interferes with the placental transport of essential amino acids, markedly reducing the activity of transport systems A, L, and y+, thereby compromising the supply of amino acids such as lysine and alanine to the fetus—particularly when combined with nicotine [152]. This interference is dose-dependent and has been documented in both placental perfusion models and microvillous vesicle assays, although some studies suggest that physiological concentrations may not significantly alter the transport of specific amino acids [153]. These disruptions in serotonin and amino acid transport may induce uteroplacental vasoconstriction, reducing blood flow and increasing the risk of hypoxia and IUGR [151].

Malek et al. [154] also reported that cocaine exposure significantly increases the release of syncytiotrophoblast-derived microparticles (MPs) into maternal circulation by approximately 30%, a phenomenon resembling alterations observed in placental oxidative stress and potentially serving as a biomarker of placental dysfunction. Concurrently, a 49% increase in placental P-glycoprotein expression was observed, which may alter the protective placental barrier and modify permeability to other xenobiotics, including therapeutic drugs.

### 5.5. Opioids

The two principal classes of opioids are morphine and its synthetic derivative heroin. While heroin is mainly known for its recreational use, morphine and other derivatives are widely prescribed as potent analgesics for various medical conditions. However, overprescription in certain countries, such as the United States, has led to a major public health issue known as opioid use disorder (OUD) [155]. According to epidemiological data, 16 million people are affected globally by OUD, with 2.1 million diagnoses reported in the USA [156]. Maternal OUD during pregnancy is a severe and growing concern rising at an alarming rate in recent years from 3.5 to 8.2 per 1000 delivery hospitalizations in the same country [157].

Agonistic and partial agonist opioids exert their effects by binding to opioid receptors, which are endogenously stimulated by endorphins—neuropeptides that modulate pain perception and induce a sense of well-being [158]. Due to their high lipophilicity and low molecular weight, opioids readily accumulate in placental tissue, posing a significant risk of neonatal dependence in infants born to opioid-using mothers [159,160].

In ex vivo placental perfusion studies, the combination of heroin and methadone significantly increases placental permeability, as measured by the passage of antipyrine [154], potentially compromising placental barrier function and facilitating the transfer of toxic substances, bacteria, or viruses to the fetus. Concurrently, heroin exposure elevates syncytiotrophoblast-derived MPs release into maternal circulation by approximately 30%, a finding similar to changes observed under placental oxidative stress, suggesting a possible biomarker of placental dysfunction. P-glycoprotein (P-gp) expression in placental tissue also increases markedly (≥49%) after heroin exposure, potentially altering the transplacental transport of other drugs [154].

At the histopathological and tissue level, chronic opioid exposure leads to loss of sensitivity and dysfunction of placental opioid receptors, as well as fibrin deposits, cellular proliferation, chronic hypoxia, infections, and cell death [161]. Morphine, in particular, inhibits the normal proliferation of CTB and STB cells, altering the structural integrity of these essential layers and disrupting hormonal secretion (notably hCG), thereby affecting the estrogen–progesterone balance and potentially compromising embryonic viability [162].

Morphine exposure also interferes significantly with nutrient transport across the placenta, impairing the formation and function of placental blood lacunae, which are crucial for maternal–fetal exchange [163]. This impairment is mediated by activation of μ, κ, and δ opioid receptors within placental villi, which inhibit adenylyl cyclase through intracellular signaling, resulting in reduced cAMP and calcium influx. Consequently, the genesis of blood lacunae is disrupted, leading to a significant reduction in both surface area and number of lacunae, particularly within the embryonic region, where vessel density and nutrient exchange are highest [163].

Heroin (diacetylmorphine), as a synthetic derivative of morphine, exhibits pharmacological characteristics that distinctly affect placental function. Due to acetylation at positions 3 and 6, heroin crosses the blood–brain barrier (BBB) much faster than morphine, greatly enhancing its lipophilicity [164]. Once administered, heroin undergoes rapid two-step deacetylation: the acetyl group at position 3 is first removed to form 6-monoacetylmorphine (6-MAM), which is then hydrolyzed at position 6 to produce morphine. Heroin also rapidly crosses the placental barrier, where it is converted into morphine, which—being less lipophilic—tends to accumulate in fetal tissues [165]. The intermediate metabolite 6-MAM has a half-life of 3–52 min and may remain detectable in plasma for several hours after heroin has been cleared [164]. Importantly, 6-MAM is a unique and specific biomarker of heroin exposure, as it is not produced from morphine or codeine [166]. Furthermore, inclusion of meconin in analytical panels and the use of high-sensitivity platforms improve discrimination between intrauterine heroin exposure and maternal codeine/morphine administration, thereby enhancing detection in exposed neonates [167].

The placenta responds to opioid exposure by modifying the activity of enzymes such as CYP19, essential in both opioid metabolism and estrogen synthesis, and by activating kappa (κ) and mu (μ) opioid receptors. Stimulation of these receptors increases the release of hCG, placental lactogen, and nitric oxide (NO), with implications for vascular regulation and endocrine function [161,168,169].

Additionally, the serotonin transporter (SERT), primarily localized to the apical STB membrane, undergoes structural alterations and the emergence of new isoforms in opioid-exposed placentas, potentially disrupting serotonin (5-HT) regulation within the intervillous space [170].

To protect the fetus, the placenta relies on active efflux transporters such as P-glycoprotein (P-gp) and BCRP, which expel xenobiotics back into the maternal circulation [170,171]. Finally, opioid exposure induces epigenetic alterations, including hypomethylation in trophoblastic cells and hypermethylation in embryonic cells, disrupting DNA regulatory regions essential for placental–fetal communication [162].

Ongoing projects such as POPI (Placenta, Opioids and Perinatal Implications) are investigating how opioid use during pregnancy promotes inflammatory changes in placental tissue and other mechanisms underlying placental dysfunction, and how these processes directly affect maternal–fetal health [172]. Pending the results obtained from such studies, accumulating evidence suggests that opioid use during pregnancy may adversely affect not only placental health but also neonatal brain development and the newborn’s capacity to respond to stress [160].

### 5.6. Other Drugs

#### 5.6.1. Methamphetamines and Amphetamines

Amphetamine (A) and methamphetamine (MA) are closely related psychostimulants that share similar mechanisms of action and placental consequences, although the addition of a methyl group in MA increases its lipophilicity, enabling it to cross the placental barrier more efficiently and thereby enhancing both its potency and toxicity [173,174].

MA is primarily metabolized in the liver by the enzyme CYP2D6, generating metabolites such as A, 4-hydroxymethamphetamine (4-OH-MA), and norephedrine. Some epidemiological studies in the U.S. have reported that approximately 5% of pregnant women recognize having consumed MA at some point of the gestation [175]. Likewise, it seems that the prevalence of pregnant women using MA have doubled between 1988 to 2004, and these women are more likely be white, unemployed, and less than 24 years old [176]. Both MA and A inhibit 5-HT and norepinephrine (NE) transporters in the human placenta, leading to increased monoamine release, vasoconstriction, and uterine stimulation within the placental space [177].

Once in maternal circulation, MA readily crosses the placenta and accumulates in fetal tissues. This accumulation has been associated with maternal and fetal hypertension, reduced uterine and umbilical blood flow, and decreased fetal oxygen and pH, all of which compromise placental functionality [178,179]. Animal studies further reveal that prenatal MA exposure decreases plasma renin responsiveness in offspring, though it does not appear to alter ACTH or prolactin levels [180].

At the histopathological level, prenatal exposure to MA has been linked to increased apoptosis, DNA oxidative damage, and focal hemorrhages in placental tissue [181,182]. However, some studies report no overt structural abnormalities in the overall placental morphology. Functionally, MA exposure leads to a reduction in placental area, giant cell number, and glycogen-positive junctional zones, as well as downregulation of genes involved in glycogen metabolism, such as glycogen synthase 1 (GYS1) and Prl7c1, impairing fetal glucose homeostasis and contributing to low birth weight (LBW) [183].

In cellular models, low concentrations of MA exhibit a protective effect on JEG-3 trophoblastic cells—which share key features with STB—by increasing nitric oxide (NO) production and reducing inflammatory responses. Conversely, high doses of MA become cytotoxic, inducing apoptosis [184].

At the molecular transport level, both A and MA compete with the serotonin transporter (SERT) and norepinephrine transporter (NET) on the apical and basal membranes of STB cells [183,184]. While cocaine primarily blocks SERT without being transported, amphetamines act as both inhibitors and substrates—they are taken up by these transporters, disrupting monoamine homeostasis inside placental cells [185,186]. This dual mechanism may explain their more sustained and intracellular effects compared to cocaine. Such alterations in transporter function can promote placental vasoconstriction, reduced nutrient exchange, and, in severe cases, placental abruption [185,186,187].

Moreover, maternal A exposure has been associated with significant changes in placental protein composition, including the appearance of new SERT isoforms and fragments and altered expression or localization of both SERT and NET throughout gestation [186]. Collectively, these findings underscore the capacity of amphetamine-type stimulants to profoundly disrupt placental signalling, vascular regulation, and metabolic function, thereby compromising foetal growth and development.

#### 5.6.2. MDMA (Ecstasy)

3,4-Methylenedioxymethamphetamine (MDMA) crosses the placenta and reaches the fetal brain, exerting direct neurotoxic effects by disrupting 5-HT and DA systems [188,189]. These effects may be mediated by several mechanisms, including direct pharmacological action, vasoconstriction, oxidative stress, and endocrine alterations such as increased cortisol levels and reduced maternal serotonin [190,191].

MDMA use has also been associated with obstetric complications such as preterm birth, IUGR, and placental abruption [192], mainly due to its vasoconstrictive properties and impact on placental function [193,194]. Furthermore, MDMA may interfere with fetal 5-HT signaling, which plays a crucial role in early embryogenesis and the development of structures such as the neural crest, limbs, and neuronal specification, potentially contributing to low birth weight [192].

Although histopathological studies in human placenta are limited, evidence from animal and in vitro models indicates that MDMA induces apoptosis, oxidative stress, and upregulation of the cellular stress response factor c-jun. These effects may be exacerbated by hyperthermia commonly associated with MDMA use [193].

In addition, MDMA can modulate the gene expression of key transporters such as RFC1, reducing folate uptake and disrupting placental function, which in turn may alter the activity of proteins and growth factors within the placenta [193,195].

Finally, although MDMA can damage serotonergic neurons through the formation of glutathione conjugates, recent studies indicate that these compounds do not inhibit glutathione-S-transferase (GST) activity in the human placenta, suggesting that the organ’s detoxification capacity remains functionally intact [196].

#### 5.6.3. New Psychotropic Drugs

New psychoactive substances (NPS) comprise a chemically diverse and rapidly evolving group of compounds designed to mimic the effects of established illicit drugs, including synthetic cannabinoids, synthetic cathinones, phenethylamines, synthetic opioids, and novel benzodiazepines [197]. Current evidence on their effects during pregnancy remains limited, heterogeneous, and difficult to interpret, owing to the rapid turnover of compounds, under-detection in routine toxicology, frequent polydrug exposure, and the scarcity of placental-specific studies [198,199]. Nevertheless, available data support the concept that NPS may affect fetal development not only through transplacental passage but also by directly altering placental structure and function.

The most detailed mechanistic evidence currently comes from the cannabinoid field. Among true NPS, synthetic cannabinoids such as JWH-018, JWH-122, and UR-144 have been shown in human trophoblast models to reduce viability, increase reactive oxygen species, disrupt mitochondrial membrane potential, and activate caspase-dependent intrinsic apoptosis, although the degree of cannabinoid receptor dependence appears to vary by compound [200]. This is an important point, as it indicates that synthetic cannabinoids should not be regarded as a mechanistically uniform class at the placental level.

Compared with synthetic cannabinoids, placental mechanistic evidence for synthetic cathinones is much more limited, but available studies still suggest biological relevance. In pregnant mice, methylone, mephedrone, and MDPV have been shown to cross the placenta and reach measurable concentrations in fetal tissues, including the fetal brain, indicating efficient transplacental passage [201]. Gestational exposure studies with mephedrone have further reported reduced maternal weight gain, decreased pup number and weight, increased stillbirth, and, in some models, placental mitochondrial and histopathological abnormalities, suggesting that developmental toxicity may involve placental compromise in addition to direct fetal injury [202,203]. A human forensic case involving α-PHP and 3,4-MDPHP is particularly noteworthy because fetal blood concentrations exceeded maternal levels, strongly supporting transplacental transfer and possible fetal accumulation, potentially through ion trapping and/or impaired fetal clearance [204]. Even so, direct molecular characterization of cathinone-induced placental injury remains largely absent.

For other NPS classes, including phenethylamines, tryptamines, synthetic opioids, and novel benzodiazepines, placental tissue-specific evidence remains very sparse. Most available human data derive from isolated case reports, targeted toxicology findings, or neonatal clinical observations rather than structured placental pathology studies [198]. This is a major limitation, particularly given that many of these compounds are likely to affect placental function indirectly through maternal vasoconstriction, altered uteroplacental perfusion, or disturbance of trophoblast signaling pathways, even when direct placental endpoints have not yet been assessed.

Human pregnancy evidence also remains difficult to interpret because NPS exposure frequently occurs in the setting of polydrug use, uncertain product composition, and incomplete biomonitoring. For example, case reports of prenatal synthetic cannabinoid exposure have described both adverse neonatal outcomes, including small-for-gestational-age birth and withdrawal-like symptoms, and apparently normal short-term outcomes, underscoring the heterogeneity of clinical presentation and the difficulty of drawing conclusions from isolated observations [205,206]. Reviews consistently emphasize that many NPS escape routine screening and require LC-MS/MS-based detection, meaning that current maternal, cord blood, and placental datasets are likely biased toward severe clinical or forensic scenarios [198].

Thus, the available literature supports a model in which NPS may compromise the maternal–placental–fetal unit through a combination of placental transfer, tissue accumulation, mitochondrial and oxidative injury, impaired trophoblast differentiation and invasion, and altered vascular or transport functions. However, mechanistic resolution remains highly uneven across drug classes, with the strongest evidence currently concentrated in cannabinoid-related compounds and far less direct placental information for cathinones and other emerging NPS. Future work will require standardized placental models, realistic exposure paradigms that account for polydrug use, and better integration of placental endpoints into developmental toxicology pipelines in order to define how these rapidly evolving substances alter placental biology and fetal risk.

### 5.7. Polysubstance Use

Polysubstance use during pregnancy is highly prevalent, frequently underestimated, and represents a major challenge for both clinical interpretation and mechanistic attribution, as exposures are often overlapping, temporally variable, and difficult to quantify with precision [16,17]. The most common combinations involve alcohol, tobacco/nicotine, and cannabis, with particularly high rates of co-use among individuals with opioid use disorder [19,207]. This clustering of exposures reflects real-world patterns and limits the interpretability of single-agent studies, highlighting the need to conceptualize prenatal drug exposure as a “mixture effect” rather than as isolated toxicological events.

Accumulating evidence indicates that polysubstance exposure can converge on shared pathways of placental dysfunction and, in some cases, produce synergistic effects on placental structure and function [18,208,209]. While the magnitude and direction of these interactions remain context-dependent, human cohort studies and experimental models suggest that combined exposures may amplify adverse outcomes through both biological convergence and pharmacokinetic interactions. For instance, dual prenatal exposure to alcohol and tobacco has been associated with an increased risk of late stillbirth, plausibly mediated by placental vascular dysfunction and uteroplacental malperfusion [210]. Similarly, population-based studies have reported that combined exposure to tobacco and cannabis is associated with altered angiogenic profiles and placental growth patterns consistent with vascular maladaptation [211].

At the molecular and cellular level, evidence from human placental studies incorporating co-exposure groups suggests that polysubstance use may perturb key regulatory hubs rather than isolated pathways. For example, placental analyses in pregnancies exposed to alcohol and/or medications for opioid use disorder have identified concurrent alterations in immune signaling and serotonin-related pathways, supporting the concept of interaction-prone systems linking inflammation, neuroendocrine regulation, and fetal programming [212]. Complementary transcriptomic and epigenetic studies further indicate that drug exposures—whether isolated or combined—target overlapping gene networks involved in angiogenesis, extracellular matrix remodeling, and neurodevelopment, reinforcing the idea of pathway convergence under co-exposure conditions [213,214].

Importantly, polysubstance interactions may also arise independently of direct molecular crosstalk at the placental level. Experimental models demonstrate that co-exposure can modify maternal drug metabolism and bioavailability, thereby increasing effective fetal and placental exposure. For example, combined exposure to alcohol and THC has been shown to increase maternal blood alcohol concentrations and alter cannabinoid metabolism, suggesting that pharmacokinetic interactions may amplify downstream placental injury [215]. This upstream mechanism is particularly relevant, as it implies that synergistic effects may emerge through altered exposure dynamics rather than exclusively through intracellular signaling interactions. Conversely, no significant differences in terms of ER stress in human placental villous explants were observed when comparing the effects of nicotine and nicotine plus ethanol [86], suggesting that some mechanisms may be specific of some drugs and independent of synergic effects between them.

From a translational perspective, it is also important to recognize that placental findings in clinical settings are rarely attributable to a single exposure. Imaging-based studies and biomarker analyses indicate that opioid-exposed pregnancies frequently involve concurrent nicotine or other substance use, with measurable effects on placental morphology and heterogeneity [216]. This underscores the need to interpret placental alterations within a multi-exposure framework, as failure to account for polysubstance use may lead to oversimplified or misleading conclusions.

Overall, polysubstance drug use during pregnancy should be understood as a biologically coherent “mixture exposure” paradigm in which placental injury reflects the integration of multiple, potentially interacting insults. These interactions may operate through convergent pathophysiological pathways, upstream pharmacokinetic modulation, or shared regulatory networks governing vascular, inflammatory, and barrier functions. However, the extent to which these effects are synergistic, additive, or antagonistic remains dependent on exposure timing, dose, and specific combinations, and is often underpowered in current human studies. Addressing this complexity will require well-characterized multi-exposure cohorts, standardized placental phenotyping, and experimental models that more accurately reflect real-world patterns of substance use.

Overall, the principal effects of the different substances on placental tissue and fetal outcomes are summarized in Table 1.

## 6. Concluding Remarks and Future Directions

This review aimed to provide an integrated overview of the effects of maternal substance use on placental structure and function, emphasizing the placenta as both a target of xenobiotic injury and a biomarker of prenatal exposure, and highlighting its clinical and forensic relevance. Overall, the evidence supports a model in which commonly used substances—including alcohol, tobacco, cannabis, cocaine, opioids, and amphetamine-type stimulants—can cross the placental barrier and induce structural and functional alterations that compromise maternal–fetal exchange and fetal development.

Key take-home messages from this review are as follows: (i) placental alterations represent a central mechanistic link between maternal exposure and adverse fetal outcomes; (ii) these alterations are multifactorial, involving vascular, oxidative, inflammatory, and endocrine pathways; (iii) the placenta provides a valuable and underutilized source of clinical and forensic information; and (iv) real-world exposure scenarios, particularly polysubstance use, significantly complicate mechanistic interpretation and risk assessment.

A major strength of this review lies in its integration of clinical, forensic, and social perspectives, providing a comprehensive understanding of how substance use during pregnancy affects placental biology and maternal–fetal health. By combining evidence from histological, molecular, and epidemiological studies, we have identified consistent patterns of placental damage that serve as biomarkers of prenatal exposure. Moreover, the discussion emphasizes the practical applications of placental analysis, such as forensic investigations, early diagnosis of fetal compromise, and planning of multidisciplinary interventions.

However, several limitations must be acknowledged. As a narrative review, this work does not aim to systematically synthesize all available evidence, and therefore may be subject to selection bias. In addition, the literature is characterized by substantial heterogeneity in study design, exposure assessment (self-report vs. biomarkers), timing, and population characteristics, which limits direct comparability across studies. Many investigations rely on retrospective or postnatal data, restricting causal inference, and placental endpoints are inconsistently assessed across experimental and clinical studies.

Importantly, although this review includes the main classes of psychoactive substance of abuse, the evidence remains limited for certain emerging substances, particularly NPS for which placental-specific mechanistic data are still scarce. More broadly, the available evidence is not always consistent across studies, reflecting differences in methodology, exposure conditions, and model systems, and highlighting that the field is still incompletely explored and requires further investigation.

These limitations underscore key methodological challenges in this area, including the difficulty of isolating single-substance effects in the context of polysubstance use, variability in analytical detection methods, and the limited translational comparability between in vitro, animal, and human studies. While many studies provide biologically plausible mechanisms, the overall evidence base remains fragmented, particularly at the level of human placental tissue.

Future research should therefore prioritize standardized and integrative approaches to better characterize placental responses to psychoactive substance exposure. In the context of NPS, this includes the development of placenta-relevant experimental models, such as trophoblast systems, ex vivo perfusion models, and placenta-on-chip platforms, combined with advanced toxicological screening methods (e.g., LC-MS/MS-based detection). Longitudinal human studies incorporating well-characterized exposure data and placental phenotyping are also needed to improve causal inference and clinical translation.

Further priorities include: (1) the study of dose–response relationships and exposure timing; (2) the effects of polysubstance use under realistic conditions; (3) the integration of molecular, histopathological, and functional placental endpoints; and (4) the evaluation of preventive and therapeutic strategies aimed at mitigating placental and fetal damage.

Overall, advancing our understanding of psychoactive substance-induced placental dysfunction will require multidisciplinary efforts bridging experimental biology, clinical research, and public health, with the ultimate goal of improving maternal–fetal outcomes in the context of substance use.

## Figures and Tables

**Table 1:** A summary of the effects of the main recreational drugs on placental tissues along with potential harms in the maternofetal wellbeing.

Drug Studied	Effects on Placental Tissue	Effects on the Fetus and Maternal–Fetal Well-Being	References
**Alcohol (Ethanol)**	- Crosses the placenta and accumulates in fetal tissues due to immature metabolism. - Induces microinfarctions, hypoxia, apoptosis, and mitochondrial and ER stress in trophoblastic cells. - Alters STB function, amino acid transport, and vascular growth factor expression (↓ PIGF, ↓ VEGFR2). - Causes oxidative stress, inflammation (↑ IL-1β, TNF-α, IL-6), and dysregulation of angiogenesis and ECM genes. - Triggers sex-dependent epigenetic alterations (↓ DNA methylation in males; ↑ SAM and TET3 in females).	- Impaired nutrient and oxygen transport leading to IUGR and fetal hypoxia. - Brain growth restriction and neurodevelopmental alterations characteristic of FASD. - Increased risk of miscarriage, low birth weight, and postnatal metabolic dysfunction. - Sex-specific adaptive responses may mitigate or exacerbate outcomes.	[69,70,71,72,73,74,75,76,77,78,79,80,81,82,83,84,85,86,87,88]
**Tobacco (Nicotine)**	- Nicotine crosses the placenta and accumulates in amniotic fluid and fetal tissues. - Causes villous hypoxia, STB hyperplasia, necrosis, excessive syncytial knots, and ER stress. - Alters expression of apoptotic (↑ BAX, TP53), inflammatory, and oxidative stress markers (↑ CYP1A1, ↑ 8-OHdG). - Dysregulates amino acid and nutrient transport (↓ hCNT1, ↓ Synb). - Induces epigenetic changes (DNA hypermethylation, ↓ miR-16, ↓ miR-21, ↓ miR-146a).	- Chronic fetal hypoxia, IUGR, and placental insufficiency. - Immune dysregulation and proinflammatory fetal phenotype (↑ IL-8). - Increased risk of preterm birth, miscarriage, and neonatal respiratory and allergic disease. - Possible lower incidence of preeclampsia via α7nAChR-mediated anti-inflammatory pathway.	[90,91,92,93,94,95,96,97,98,99,100,101,102,103,104,105,106,107,108,109,110,111,112,113,114,115,116]
**Cannabis (THC, CBD, minor cannabinoids)**	- Disrupts endocannabinoid system homeostasis essential for placental development. - Induces mitochondrial dysfunction (↓ HSP60/HSP70, ↑fission markers), ER stress (↑ CHOP, PERK, IRE1), and apoptosis in trophoblasts. - Alters hormone synthesis (↓ hCG, ↓ leptin, ↓ hPL) and glucose transporter expression (↓ GLUT1, ↑ GLUT3). - Modifies protein composition (↓ CYP19, ↓ BCRP) and increases placental permeability. - CBD impairs angiogenesis (↓ VEGFA, ↓ PGF) and vascular formation; THC and CBD trigger inflammatory cytokine release (↑ IL-1β, IL-6, TNFα). - Epigenetic effects: altered DNA methylation at >500 CpG sites (notably *MEGF10* hypermethylation).	- Impaired maternal–fetal blood and nutrient exchange. - Increased risk of IUGR, placental insufficiency, and preterm delivery. - Neurodevelopmental alterations and increased risk of cognitive and behavioral disorders in offspring. - Potential transgenerational epigenetic consequences.	[120,121,122,123,124,125,126,127,128,129,130,131,132,133,134,135]
**Cocaine**	- Potent vasoconstrictor: blocks catecholamine reuptake → ↑ adrenaline/noradrenaline, ↑ endothelin, ↓ NO → marked placental vasospasm and ischemia. - Structural vascular damage: endothelial injury, ↑ vascular permeability, leukocyte adhesion, platelet aggregation. - Accumulates in placental tissue (lipophilic, low MW) → prolonged local toxicity. - Induces oxidative stress (↓ catalase, SOD, GPx → ↑ ROS, lipid peroxidation) and apoptosis (↑ caspase-3, Bax/Bcl-2 imbalance). - Inhibits placental serotonin and norepinephrine transporters (SERT, NET); ↓ amino acid transport systems A, L, y+. - ↑ Syncytiotrophoblast microparticle release (~30%) and ↑ P-glycoprotein expression (~49%) as markers of stress and altered barrier function.	- Decreased placental perfusion → hypoxia, IUGR, fetal distress, preterm delivery. - Possible villous hemorrhage and edema. - Sustained fetal exposure due to cocaine and metabolite accumulation (benzoylecgonine, norcocaine). - ↑ Risk of placental abruption, miscarriage, and perinatal mortality. - Neurodevelopmental deficits related to chronic hypoxia and monoaminergic disruption.	[138,139,140,141,142,143,144,145,146,147,148,149,150,151,152,153,154]
**Opioids (Morphine, Heroin, Methadone)**	- Readily cross placenta; accumulate due to high lipophilicity. - ↑ Placental permeability (↑ antipyrine transfer), ↑ MP release (~30%), ↑ P-gp expression (~49%). - Histopathology: fibrin deposits, villous proliferation, chronic hypoxia, infection, apoptosis. - Inhibits CTB/STB proliferation and hCG secretion → hormonal imbalance (↓ estrogen/progesterone ratio). - Interferes with formation of placental lacunae (μ, κ, δ receptor activation → ↓ cAMP, ↓ Ca^2+^ influx). - Modifies CYP19 activity, alters SERT structure, induces DNA hypo-/hypermethylation in trophoblastic and embryonic cells. - Ongoing evidence of inflammatory and vascular remodeling (POPI project).	- Neonatal abstinence syndrome; impaired neurodevelopment and stress response. - IUGR, preterm birth, hypoxia-related complications. - Altered vascular regulation (NO, hCG, placental lactogen changes). - Potential transgenerational epigenetic effects on placental–fetal communication.	[158,159,160,161,162,163,164,165,166,167,168,169,170,171]
**Amphetamines/** **Methamphetamines**	- Inhibit placental 5-HT and NE transporters → ↑ monoamine release, vasoconstriction, uterine stimulation. - Cross placenta easily; accumulate in fetal tissues → ↓ uterine/umbilical blood flow, ↓ O_2_ and pH. - Induce apoptosis, oxidative DNA damage, and focal hemorrhage in placenta. - ↓ Placental area, ↓ giant cells, ↓ glycogen zones; ↓ GYS1 and Prl7c1 expression → impaired glycogen metabolism. - Dose-dependent cytotoxicity: low doses ↑ NO (protective), high doses → apoptosis. - Compete with SERT/NET as substrates (not just inhibitors) → sustained intracellular effects. - Alter SERT and NET isoforms and localization throughout gestation.	- Fetal growth restriction, LBW, placental insufficiency. - Preterm birth and increased risk of placental abruption. - Fetal hypoxia and metabolic dysregulation due to altered glucose and amino acid homeostasis. - Long-term neurodevelopmental deficits and cardiovascular dysregulation in offspring.	[177,178,179,180,181,182,183,184,185,186]
**MDMA (Ectasy)**	- Crosses placenta, reaching fetal brain → direct neurotoxicity via disrupted 5-HT and DA systems. - Mechanisms: direct pharmacological action, vasoconstriction, oxidative stress, ↑ cortisol, ↓ maternal serotonin. - Induces apoptosis, oxidative stress, ↑ c-jun transcription; potentiated by hyperthermia. - ↓ RFC1 expression → ↓ folate uptake → altered placental protein/growth factor activity. - Does not impair GST detoxification function despite glutathione conjugate formation.	- Obstetric complications: preterm birth, IUGR, placental abruption. - Interference with fetal 5-HT signaling → abnormal embryogenesis (neural crest, limb, neuronal development). - LBW and potential neurobehavioral alterations. - Sustained risk from combined endocrine and oxidative stress effects.	[192,193,194,195,196]
**New Psychoactive substances**	- Cross the placenta and may accumulate in fetal tissues; many compounds are underdetected in routine toxicology. - Synthetic cannabinoids (e.g., JWH-018, JWH-122, UR-144) reduce trophoblast viability, increase ROS, disrupt mitochondrial membrane potential, and activate caspase-dependent intrinsic apoptosis. - Likely impair trophoblast differentiation, invasion, and vascular/transport functions; mechanistic evidence is strongest for cannabinoid-related NPS. - Synthetic cathinones show efficient transplacental passage and probable placental mitochondrial and histopathological injury, although direct molecular placental evidence remains limited. - Mechanistic heterogeneity across compounds is high; placental effects are not uniform within the NPS group.	- Fetal exposure may be substantial, including detectable concentrations in fetal brain for some cathinones. - Associated with reduced pup number/weight, stillbirth, fetal growth impairment in animal models and possible withdrawal-like neonatal symptoms in reported human cases. - Clinical interpretation is limited by polydrug exposure, uncertain compound composition, and incomplete biomonitoring. - Potential adverse outcomes include SGA, fetal accumulation, neurotoxicity, and impaired maternal–placental–fetal exchan	[198,199,200,201,202,203,204,205,206]
**Polydrug use**	- Commonly involves overlapping exposure to alcohol, tobacco/nicotine, cannabis, and opioids, limiting single-agent interpretation. - Promotes convergent placental injury through shared vascular, inflammatory, oxidative, endocrine, and transport-related pathways. - May produce additive or synergistic effects on placental structure and function, including uteroplacental malperfusion and vascular maladaptation. - Alters key regulatory hubs such as immune signaling, serotonin-related pathways, angiogenesis, extracellular matrix remodeling, and epigenetic programming. - Pharmacokinetic interactions may increase effective maternal, placental, and fetal exposure even without direct intracellular synergy.	- Increased risk of adverse outcomes such as late stillbirth, IUGR, placental insufficiency, and fetal growth impairment. - Clinical and forensic interpretation is more complex because placental lesions often reflect multiple simultaneous exposures. - Real-world multi-exposure patterns may amplify fetal risk beyond that predicted from isolated substances. - Requires multi-exposure interpretation to avoid oversimplified conclusions regarding fetal and neonatal outcomes.	[18,86,208,209,210,211,212,213,214,215,216]

↓: downregulation or decrease; ↑: upregulation or increase.

## Data Availability

No new data were created or analyzed in this study. Data sharing is not applicable to this article.

## Abbreviations

5-HT	Serotonin
8-OHDG	8-hydroxyguanosine
A(s)	Amphetamine(s)
BCRP	Breast Cancer Resistance Protein
CBD	Cannabidiol
CNS	Central nervous system
CTB	Cytotrophoblasts
CYP19	Aromatase
DA	Dopamine
ECS	Endocannabinoid System
eEVT	Endovascular Extravillous Trophoblast
EVT	Extravillous Trophoblast
FASD	Fetal Alcohol Spectrum Disorder
GST	Glutathione-S-transferase enzyme
GYS1	Glycogen Synthase 1
hCG	Human Chorionic Gonadotropin
iEVT	Interstitial Extravillous Trophoblast
IUGR	Intrauterine Growth Restriction
LDL	Low-Density Lipoproteins
MA	Methamphetamine
MDMA	3,4-Methylenedioxymethamphetamine
MVM	Maternal Vascular Malperfusion
NAS	Neonatal Abstinence Syndrome
NE	Norepinephrine
NET	Norepinephrine Transporter
NO	Nitric Oxide
P-gp	P-glycoprotein
PGF	Placental Growth Factor
PP13	Placental Protein 13
SAM	S-adenosylmethionine
SERT	Serotonin Transporter
SNS	Sympathetic Nervous System
STB	Syncytiotrophoblast
THC	Tetrahydrocannabinol
